# Enhanced antitumor immune responses via a new agent [^131^I]-labeled dual-target immunosuppressant

**DOI:** 10.1007/s00259-022-05986-4

**Published:** 2022-10-15

**Authors:** Chunjuan Jiang, Qiwei Tian, Xiaoping Xu, Panli Li, Simin He, Jian Chen, Bolin Yao, Jianping Zhang, Ziyi Yang, Shaoli Song

**Affiliations:** 1 Department of Nuclear Medicine, Fudan University Shanghai Cancer Center, Shanghai, China; 2 Department of Nuclear Medicine, The Second Xiangya Hospital, Central South University, Changsha, China; 3 Shanghai Engineering Research Center of Molecular Imaging Probes, Shanghai, China; 4 Shanghai Key Laboratory of Molecular Imaging, Shanghai University of Medicine and Health Sciences, Shanghai, China; 5 Key Laboratory of Smart Drug Delivery, Ministry of Education, School of Pharmacy, Fudan University, Shanghai, China

**Keywords:** Iodine-131, Immunotherapy, Immune susceptibility, Checkpoint blockade, Melanoma

## Abstract

**Abstract:**

Radionuclides theranostic are ideal “partners” for bispecific antibodies to explore the immune response of patients and synergistic treatment. A bispecific single-domain antibody-Fc fusion protein, KN046, exhibits a good treatment effect by binding to programmed cell death-ligand 1 (PD-L1) and cytotoxic T-lymphocyte-associated protein 4 (CTLA-4). An ionizing-radiation stimulus mediated by a low-dose of [^131^I] may be used for immunopotentiation. In this study, we established [^131^I]-labeled KN046 as a novel radioimmunotherapy agent to treat malignant melanoma and explored the mechanism.

**Methods:**

After intravenous injection of [^131^I]-KN046, SPECT/CT imaging was applied to identify candidate targets for KN046 immunotherapy. [^18^F]-FDG and [^68^ Ga]-NOTA-GZP (granzyme B-specific PET imaging agent) micro-PET/CT imaging was used to assess the immune response in vivo after [^131^I]-KN046 treatment. The synergistic treatment effect of [^131^I]-KN046 was evaluated by exploring the [^131^I]-based radionuclide-induced release of tumor immunogenicity-related antigens as well as the histology and survival of tumor-bearing mice after treatment.

**Results:**

The constructed [^131^I]-KN046 exhibited high affinity and specificity for PD-L1/CTLA-4 immune targets and had excellent in vivo intratumoral retention capability so as to achieve good antitumor efficacy. More importantly, the combination of low-dose [^131^I] and KN046-enhanced immunosensitivity increased the immunotherapy response rates significantly. Exposure of tumor cells to [^131^I]-KN046 led to upregulated expression of MHC-I and Fas surface molecules and significant increases in the degree of T-cell activation and counts of tumor-infiltrating immunocytes.

**Conclusion:**

Use of low-dose [^131^I] combined with a dual-target immunosuppressant could be exploited to identify the subset of treatment responders but also exhibited great potential for enhancing antitumor immune responses.

**Supplementary Information:**

The online version contains supplementary material available at 10.1007/s00259-022-05986-4.

## Introduction

Immunotherapy based on immune-checkpoint blockers (ICBs), especially combination of therapies targeting programmed cell death inhibitor 1 (PD-1) and its ligand (programmed cell death ligand 1, PD-L1) and cytotoxic T-lymphocyte-associated protein 4 (CTLA-4), has made a significant impact on the outcome of malignant melanoma [1–3]. For example, Opdivo + Yervoy dual immunotherapy in first-line treatment of advanced melanoma has been reported to have long-term survival benefit because 53% of patients remained alive at follow-up 4 years later [4]. However, dual immunotherapy is not only expensive; it also leads to significant increases in therapeutic toxicity and drug resistance [5]. In a clinical trial receiving combination therapy, 55% of patients reported grade 3 or 4 immune-related adverse events (irAE) [6]. To address these limitations, bispecific simplified fusion antibodies have been developed for treatment of malignant melanoma. Compared with dual immunotherapy, programmed cell death-ligand 1 (PD-L1)/CTLA-4 bispecific single-domain antibody-Fc fusion proteins such as KN046 can exhibit good treatment effects by binding to PD-L1 and CTLA-4 while simultaneously reducing the side effects and treatment costs due to their simplified structure [7]. Despite the important advantages of bispecific fusion antibodies, there still an urgent need for the strategies to identify the candidate patients who will benefit from PD-L1/CTLA-4 immunotherapy and increase immune response rates.

Radionuclides theranostic are a promising platform for nuclear medical imaging and cancer treatment [8–10]. They are ideal “partners” for bispecific fusion antibodies to explore the immune response of patients and synergistic treatment. Nuclear medical imaging is being employed increasingly to assess the efficacy of drugs in vivo noninvasively [11–14]. In our previous study, we reported [^68^ Ga]-labeled granzyme B PET/CT imaging which closely associated with granzyme B expression could noninvasively detect intertumoral immune response state [7]. In addition, emerging evidence has demonstrated that radionuclide therapy can induce the release of tumor immunogenicity-related antigens, resulting in synergistic enhancement of ICB therapy [15, 16]. For instance, [^213^Bi] [17] and [^177^Lu] [18] combined with anti-PD-1 antibody have been shown to be more effective in significantly increasing animal viability and retarding tumor growth than use of an ICB alone or radionuclide therapy alone. However, radionuclide labeling (e.g., [^177^Lu]) conditions are complicated, and protein denaturing can occur. In addition, the radionuclide and immune antibody must be administered separately. This action results in nonspecific distribution of nuclides to elicit significant toxic effects to normal tissue, and cannot be used directly to evaluate the response efficiency of antibodies. Therefore, developing a method to obtain a radionuclide-labeled bispecific fusion antibody with normal activity to noninvasively investigate the response efficiency and effect of synergistic treatment remains a great challenge.

[^131^I] is an important radionuclide. It can be employed for clinical diagnoses [19] based on single-photon emission-computed tomography (SPECT/CT) and for radionuclide therapy [20]. Based on modest ionizing radiation, expression of various tumor antigens in a tumor microenvironment (TME) can also be upregulated [21], which can be used for synergistic enhancement of ICB therapy. For example, [^90^Y] has been shown to increase expression of immune-susceptibility markers, such as major histocompatibility complex I (MHC-I) and FS-7-associated surface antigen (Fas) [22]. Recognition of T cells is reliant on the aid of such molecules, so upregulated expression of immune-associated antigens could improve tumor immunogenicity [23, 24]. More importantly, [^131^I] can be labeled readily on a protein via the catalyst iodogen [25], which does not affect protein activity and interfere with the normal function of target immune cells [26]. Therefore, [^131^I]-labeled ICB agents can be used to help selecting potential responders to the immunotherapy.

**Fig. 1:**
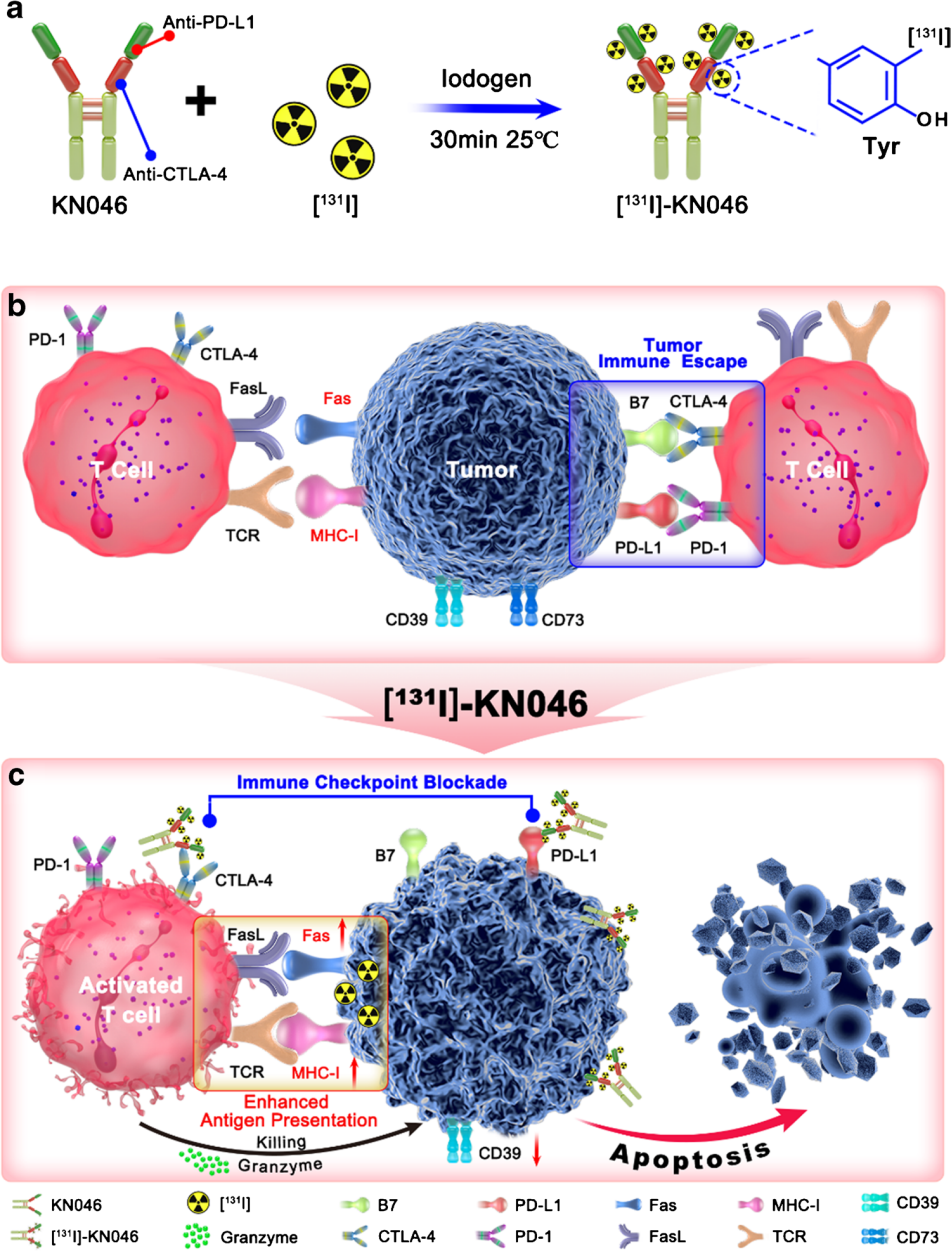
Use of [^131^I]-KN046 for noninvasive assessment and enhanced antitumor immune responses (schematic). **a** Preparation of [^131^I]-KN046. KN046 was labeled with [^131^I] using the catalyst iodogen. **b** Mechanism of tumor immune escape in the tumor microenvironment (TME). PD-L1 and B7 molecules expressed on the surface of tumor cells inhibit the activity and function of T cells by specific binding with PD-1 and CTLA-4 on the surface of T-cell membranes. **c** [^131^I]-KN046 exerts enhanced antitumor efficacy after intravenous administration. KN046 not only exhibits a good treatment effect by binding to PD-L1 and CTLA-4, the enhanced enrichment of [^131^I] due to targeting of KN046 can upregulate expression of MHC-I and Fas to stimulate the effector functions of activated T cells for cancer immunotherapy

As a proof of concept, [^131^I]-labeled KN046 was established as a novel agent to noninvasively assess the response efficiency of KN046 to malignant melanoma and synergistic effect of radiotherapy-induced modulation of the immune microenvironment by dual-targeted immunotherapy (Fig. 1a). We demonstrated, for the first time, that [^131^I] could be labeled on KN046 effectively and that targeted activity of KN046 was not affected after labeling with [^131^I]. Then, using [^131^I]-KN046 radioimmunotherapy drug, the tumor cell-targeting capacity of KN046 was investigated by [^131^I]-based SPECT/CT in vivo and in vitro, which could be used to noninvasively assess the tumor response to KN046. After that, the synergistic treatment effect of [^131^I]-KN046 was evaluated by exploring the [^131^I]-based radionuclide-induced release of tumor immunogenicity-related antigens as well as the histology and survival of tumor-bearing mice after treatment (Fig. 1b and c). The developed [^131^I]-KN046 addressed the issue of identifying the subset of treatment responders, but also exhibited great potential for enhancing antitumor immune responses.

## Results

### Labeling yield and stability

**Fig. 2:**
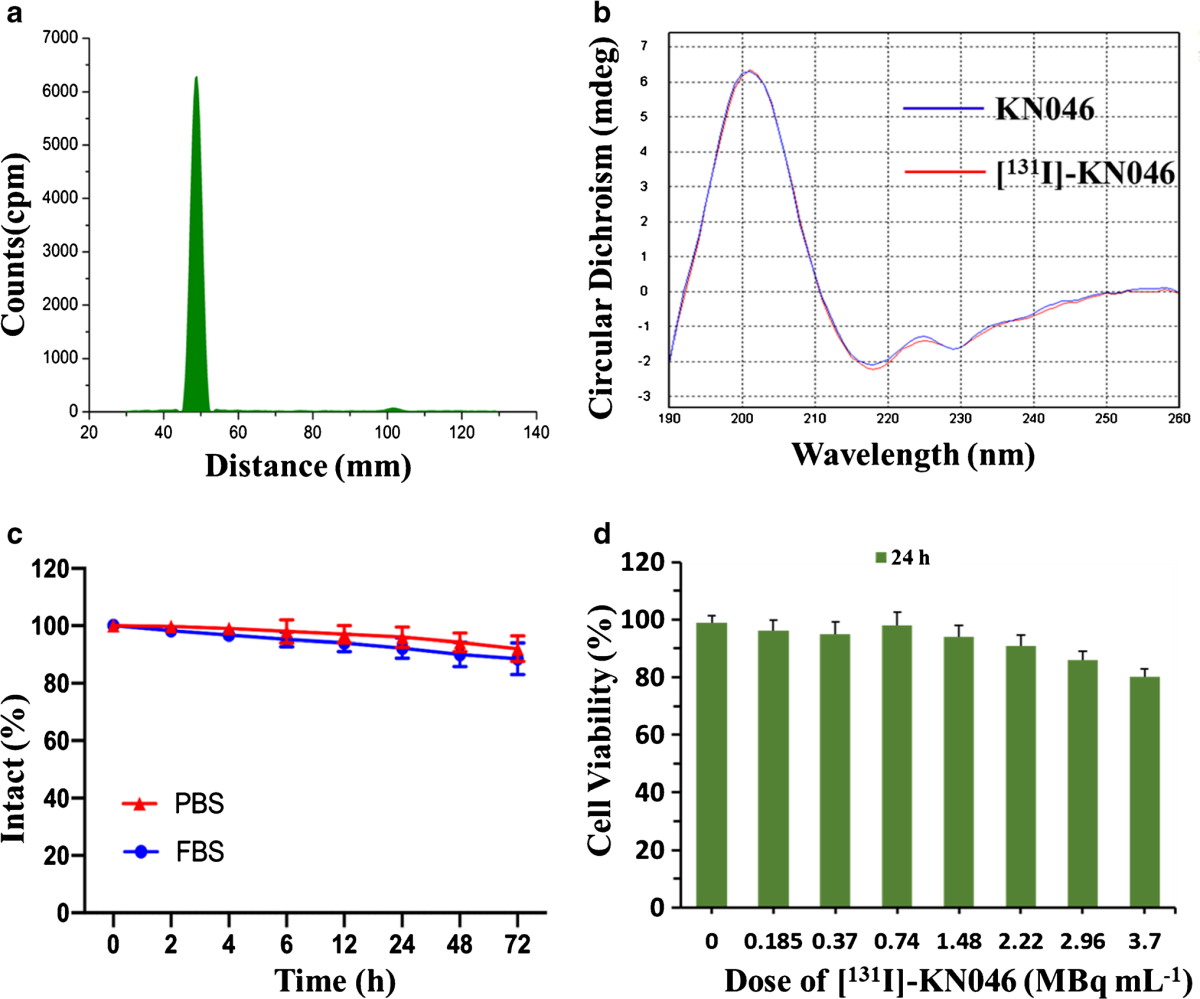
**a** Initial labeling yield of [^131^I]-KN046 placed for 30 min at room temperature. **b** Circular dichroism (CD) spectra of KN046 and [^131^I]-KN046. **c** Radiolabeling stability of [^131^I]-KN046 co-incubated in 10% FBS or PBS over time. Data are the mean ± SD. **d** Viability of B16F10 cells treated with [^131^I]-KN046 (under an [^131^I] dose of 0, 0.185, 0.37, 0.74, 1.48, 2.22, 2.96 and 3.7 MBq/mL, respectively) for 24 h. Data are the mean ± SD (*n* = 5)

The initial labeling yield of [^131^I]-KN046 was 99.99% (Fig. 2a), which indicated that it was successfully radiolabeled and can be used in the next experiment without purification. Also, the circular dichroism (CD) spectra of KN046 and [^131^I]-KN046 indicated that the targeted activity of KN046 was not affected after labeling with [^131^I] (Fig. 2b). During 72-h incubation of [^131^I]-KN046 with PBS and FBS, the labeling yield remained > 85% (Fig. 2c), indicating that [^131^I]-KN046 was stable in the in vitro-simulated TME.

### CCK-8 assay

We studied the effects of different doses of [^131^I]-KN046 on cells using the CCK-8 assay. [^131^I]-KN046 at an [^131^I] dose < 3.7 MBq had no notable toxicity to B16F10 cells and was not a lethal dose (Fig. 2d). Therefore, 1.85 MBq and 3 × 1.85 MBq of [^131^I]-KN046 were sublethal when treating a murine melanoma cell line (B16F10).

### Cellular uptake of KN046

**Fig. 3:**
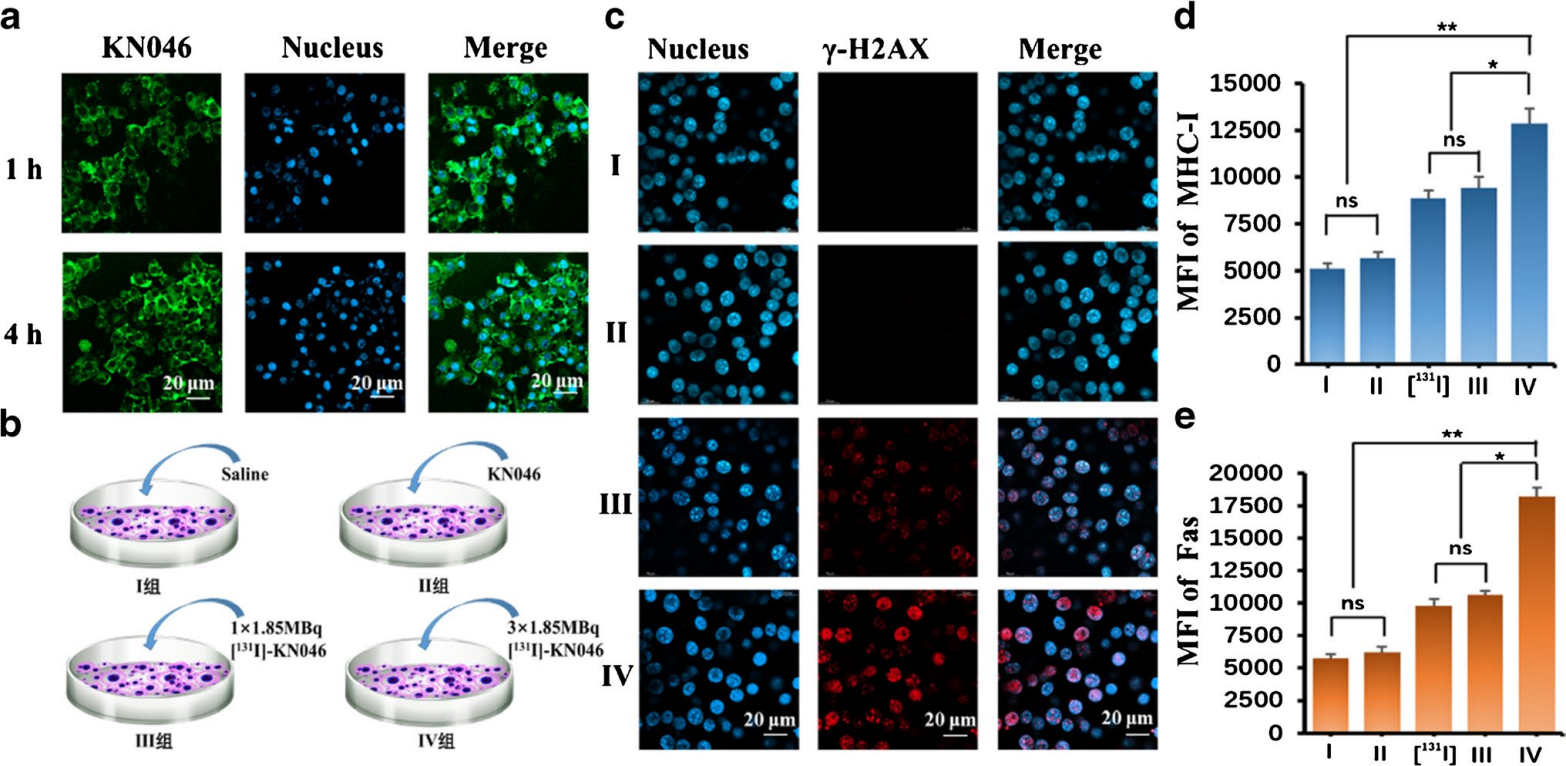
**a** Cell uptake of KN046 (green) co-incubated with B16F10 cells at 1 h and 4 h. **b** Design of the therapy protocol in vitro. **c** Representative immunofluorescence micrographs of γ-H2AX expression in B16F10 cells after different treatments. MFI of MHC-I (**d**) and Fas (**e**) after different treatments, respectively. Nuclei were stained with 4′,6-diamidino-2-phenylindole (DAPI) (blue). Data are the mean ± SD (*n* = 5). *MFI*, mean fluorescence intensity. ^*^p < 0.05, ^**^p < 0.01, *ns* no significance

The results of immunohistochemistry (IHC) showed that the PD-L1 expression on the membranes of B16F10 cells was strongly positive. However, the MCF-7 cells were PD-L1-negative expression (Fig. S1). Confocal laser scanning microscopy (CLSM) of FITC-labeled KN046 after co-culture with B16F10 cells for 1 h showed that a large amount of green fluorescence protein-labeled KN046 was taken up at the cell-membrane surface. As the incubation time extended to 4 h, the mean fluorescence intensity (MFI) of cell membrane-bound KN046 increased compared with that at 1 h (Fig. 3a). But the MFI of KN046 observed on MCF-7 cells at 1 h and 4 h was negligible compared to B16F10 cells (Fig. S2).

### Cellular gamma histone 2AX (γ-H2AX) expression after [^131^I]-KN046 therapy

As a radiation “bio-dosimeter,” γ-H2AX is more sensitive and reliable than other intracellular proteins capable of representing DNA damage/repair [27]. B16F10 cells were divided into four groups: I, physiologic (0.9%) saline; II, KN046; III, one fraction of [^131^I]-KN046; and IV, three fractions of [^131^I]-KN046 (Fig. 3b). Physiologic saline alone and KN046 alone did not cause DNA damage (Fig. 3c). However, one fraction of 1.85 MBq radiation enabled B16F10 cells to elicit DNA damage, and three fractions of 1.85 MBq radiation exacerbated that damage.

### Phenotype regulation of proteins in tumor-cell membranes after [^131^I]-KN046 treatment

After cytotoxic T lymphocytes (CTLs) recognize target cells, binding of Fas ligand to Fas on the tumor cell surface triggers an apoptotic program within tumor cells [28]. MHC-I antigen on tumor cell surfaces can present pathogenic peptides to activated CTLs to kill target cells [29]. Under normal conditions, tumor cells will downregulate expression of such protein molecules to escape recognition by immunocytes [30]. We showed that, after B16F10 cells had been exposed to [^131^I]-KN046, the MFI of the membrane proteins MHC-I and Fas increased from 5094 to 9494 and from 5731 to 10,593, respectively (Fig. 3d, e, and S3). Under an identical dose of [^131^I] radiation, the MFI increase expressed by MHC-I and Fas classes was similar to that by [^131^I]-KN046, whereas “spiking” with KN046 alone did not result in a significant increase in the MFI expressed by MHC-I and Fas classes. Melanoma cells received three fractions of [^131^I]-KN046 radiation: the MFI of MHC-I and Fas increased significantly compared with that after one-fraction radiation. The data shown above indicated that exposure of B16F10 cells to [^131^I]-KN046 or [^131^I] resulted in upregulated expression of MHC-I and Fas, and that expression of MHC-I and Fas increased in a dose-dependent manner, which is in accordance of data from other studies [22, 31, 32].

### SPECT/CT imaging, pharmacokinetics, and biodistribution of [^131^I]-KN046

**Fig. 4:**
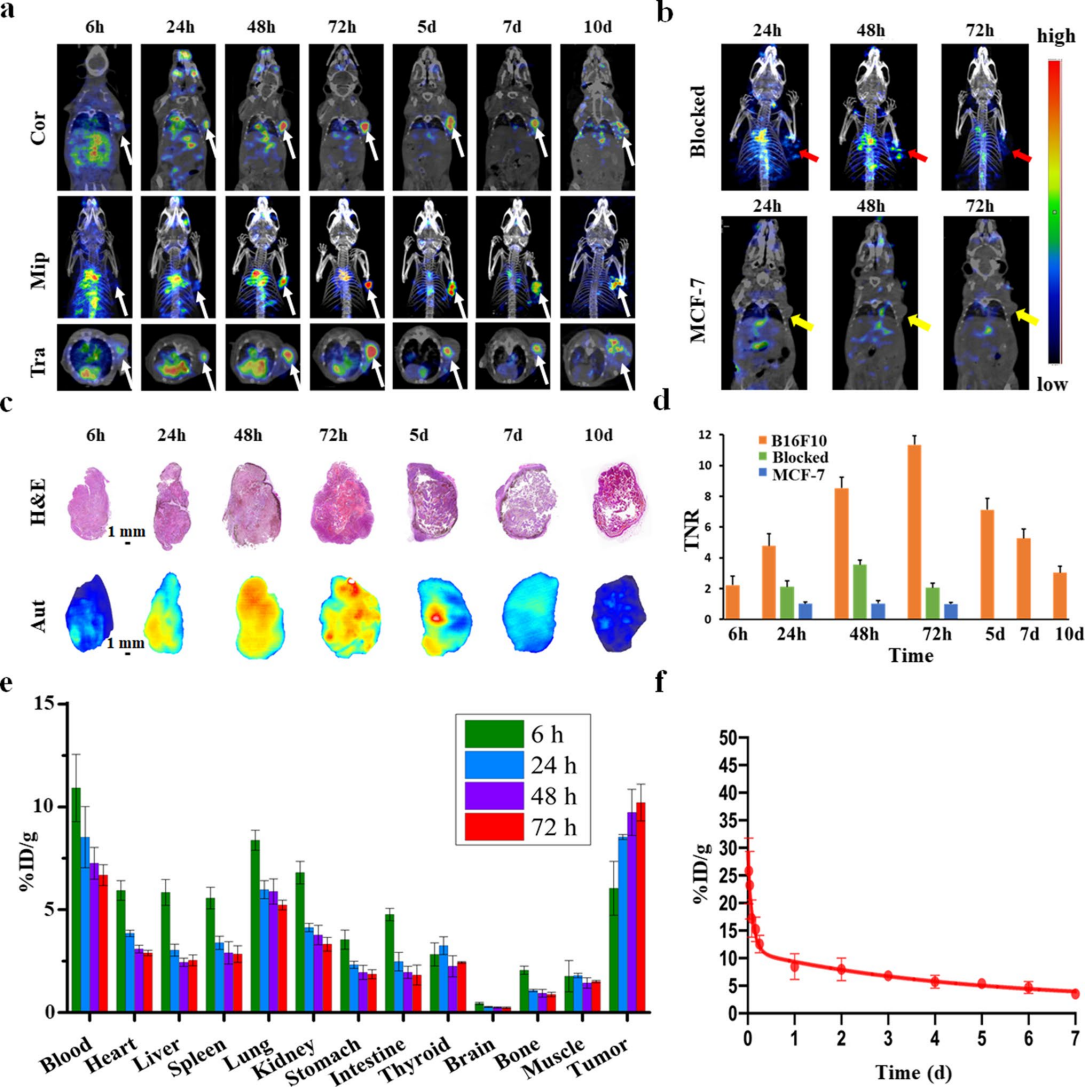
**a** Representative SPECT/CT in mice bearing B16F10 tumors at different times following a single intravenous injection of [^131^I]-KN046 ([^131^I]: 7.4 MBq, KN046: 100 μg). Top row, coronal images. Second row, maximum intensity projection (Mip). Bottom row, transverse images of tumors. **b** Representative SPECT/CT of blockade group (top row) in rats bearing B16F10 tumors and control group (bottom row) in rats bearing MCF-7 tumors at 24, 48, and 72 h after intravenous injection of [^131^I]-KN046. **c** Hematoxylin and eosin (H&E) staining (top row) and corresponding autoradiograms (Aut) (bottom row) of B16F10 tumors at different times following intravenous injection of [^131^I]-KN046. **d** Target-to-normal tissue ratio (TNR) of SPECT/CT in B16F10 tumor group, blockade group, and MCF-7 tumor group was calculated for comparison. **e** Biodistribution of [^131^I]-KN046 in mice bearing B16F10 tumors at 6, 24, 48, and 72 h post-injection. **f** Pharmacokinetics of [^131^I]-KN046 in normal BALB/c mice over time. % ID/g tissue, percentage injected dose per gram of tissue. Data are the mean ± SD (*n* = 5)

This study included two series of experiments in vivo for the assessment of [^131^I]-KN046. The first series was to study the distribution and metabolism of [^131^I]-KN046. Systemic SPECT/CT of B16F10 tumor-bearing mice demonstrated high uptake of [^131^I]-KN046 in melanoma cells (Fig. 4a). Higher TNRs could be observed (4.77 ± 0.81) 24 h after a single intravenous injection of [^131^I]-KN046, and the maximum TNR (11.34 ± 0.62) was achieved 72 h post-injection and then the TNR tapered over time (Fig. 4d). Except for slight retention in the blood system, a large part of the injected [^131^I]-KN046 accumulated and was retained in tumor tissue, and a single injection of [^131^I]-KN046 could sustain imaging until day 10 (Fig. 4a and d). In stark contrast, [^131^I]-KN046 uptake in the blockade group declined markedly, whereas residence or uptake of [^131^I]-KN046 was barely observed in tumor-bearing MCF-7 mice (PD-L1-negative expression) (Fig. 4b and d). Images from in vitro autoradiography demonstrated the metabolism of [^131^I]-KN046 in B16F10 tumor tissues (Fig. 4c). Intratumoral uptake of [^131^I]-KN046 remained high at 48 h and 72 h, peaking at 72 h, which corroborated SPECT/CT results. Hematoxylin and eosin (H&E) staining revealed a necrotic region that appeared gradually at the center of the tumor 3 days after treatment with [^131^I]-KN046 (Fig. 4c).

Next, the biodistribution profiles of [^131^I]-KN046 in B16F10 tumor-bearing mice were acquired by ex vivo gamma counting of various organ tissues harvested from sacrificed mice at different time points (Fig. 4e). The results agreed with the data observed in SPECT/CT and autoradiography. Six hours after intravenous injection, [^131^I]-KN046 was concentrated primarily in blood (12.12 ± 1.64%ID/g) (Fig. 4f) and blood-rich organs, such as the heart, liver, and spleen. At 24 h, uptake of [^131^I]-KN046 in the blood, heart, liver, and spleen decreased, whereas tumor uptake increased to 8.54 ± 0.13%ID/g. At 48 h and 72 h post-injection, uptake of [^131^I]-KN046 in each organ continued to decline, whereas its uptake in tumors increased continuously to 9.73 ± 1.12%ID/g and 10.21 ± 0.89%ID/g, respectively. Therefore, [^131^I]-KN046 in non-target tissues was cleared gradually at 24 h through 72 h, whereas its uptake in tumor tissue accumulated continuously.

### Antitumor efficacy and immune response using [^68^ Ga]-NOTA-GZP combined with [^18^F]-FDG micro-PET/CT

The second series of this study was to assess the intratumoral immune response and tumor metabolism after [^131^I]-KN046 treatment in real time. Two kinds of detection technique, [^18^F]-FDG micro-PET/CT in vivo combined with [^68^ Ga]-NOTA-GZP micro-PET/CT, were employed (Figs. S4a and 5a). Granzyme B-targeted [^68^ Ga]-NOTA-GZP was synthesized by our research team [7]. As shown by the results of [^18^F]-FDG micro-PET (Fig. S4b), after completion of three-fraction [^131^I]-KN046 treatment, tumor maximum standard unit value (SUV_max_) decreased, and differences in the treatment group between time points were small, but a reduction in the TNR was observed (*p* < 0.05) (Fig. S4c). These findings indicated that tumor metabolism in mice declined after treatment with [^131^I]-KN046, but throughout treatment, [^18^F]-FDG micro-PET/CT failed to reflect the intratumoral immune response.

**Fig. 5:**
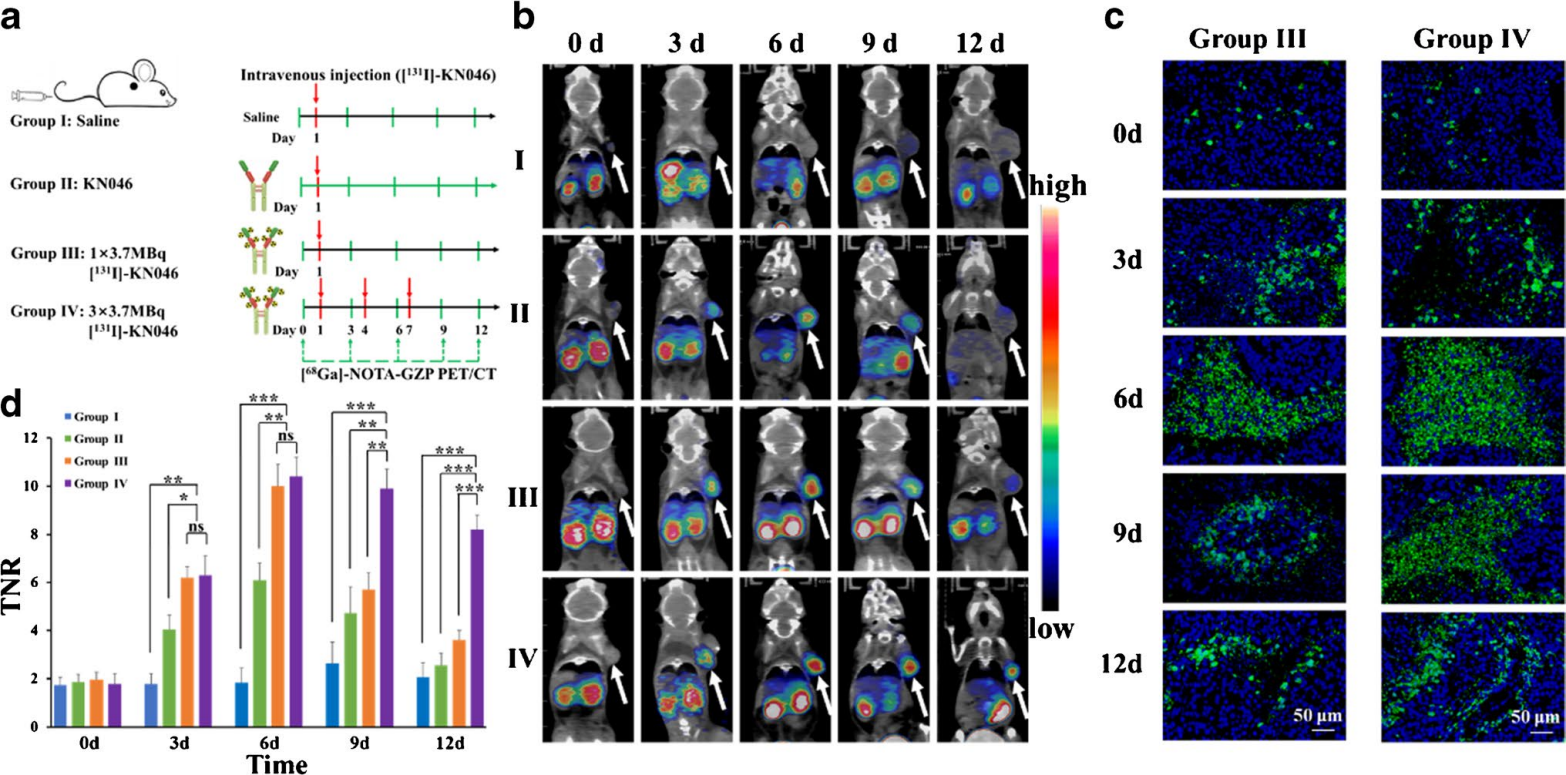
**a** Therapy protocol and [^68^ Ga]-NOTA-GZP micro-PET/CT in mice bearing B16F10 tumors (group I, saline; group II, one dose of KN046 (1 mg); group III, one fraction of [^131^I]-KN046 ([^131^I]: 1 × 3.7 MBq, KN046: 1 mg); group IV, three fractions of [^131^I]-KN046 ([^131^I]: 1 × 3.7 MBq, KN046: 1 mg) at days 1, 4, and 7. [^68^ Ga]-NOTA-GZP micro PET/CT was performed at days 0, 3, 6, 9, and 12). **b** Representative [^68^ Ga]-NOTA-GZP micro-PET/CT images in mice bearing B16F10 tumors at different times after different treatments. White arrows indicate tumors. **c** Representative immunofluorescence micrographs of caspase-3 in B16F10 tumors at different times after different treatments. Cell nuclei are stained with DAPI (blue). **d** TNR quantitative analysis of images in B16F10 tumors collected 3 days after different treatments (*n* = 5). ^*^p < 0.05, ^**^p < 0.01, ^***^p < 0.001, *ns* no significance

[^68^ Ga]-NOTA-GZP PET/CT (Fig. 5b) showed that, starting at day 3, a difference in intratumoral expression of granzyme B occurred in the treatment group versus the control group. After treatment with [^131^I]-KN046 versus use of KN046 alone, intratumoral [^68^ Ga]-NOTA-GZP uptake increased significantly (*p* < 0.05), and the TNR peaked at day 6 after one fraction [^131^I]-KN046 treatment and then tapered until day 12 (Fig. 5b and d). Imaging after treatment with three consecutive fractions of [^131^I]-KN046 revealed that intratumoral [^68^ Ga]-NOTA-GZP uptake increased continuously until day 12, when high uptake could be observed (Fig. 5b and d). CTL killing can be achieved via perforin and granzyme B. Granzyme B activates the caspase cascade [33] to lead to apoptosis. Immunofluorescence staining of caspase 3 after [^131^I]-KN046 treatment demonstrated expression of granzyme B. This result also showed the same trend as that of [^68^ Ga]-NOTA-GZP uptake at the tumor site (Fig. 5c). Hence, [^68^ Ga]-NOTA-GZP PET/CT demonstrated convincingly the real-time status of intratumoral immune activation before and after [^131^I]-KN046 therapy. Notably, mice receiving [^131^I]-KN046 therapy exhibited higher intratumoral expression of granzyme B than mice receiving KN046 therapy alone, which boosted immunotherapy efficacy. Meanwhile, higher immunotherapy response rates and a persistent antitumor immune effect were observed in mice receiving three fractions of [^131^I]-KN046.

### *In vivo *efficacy

**Fig. 6:**
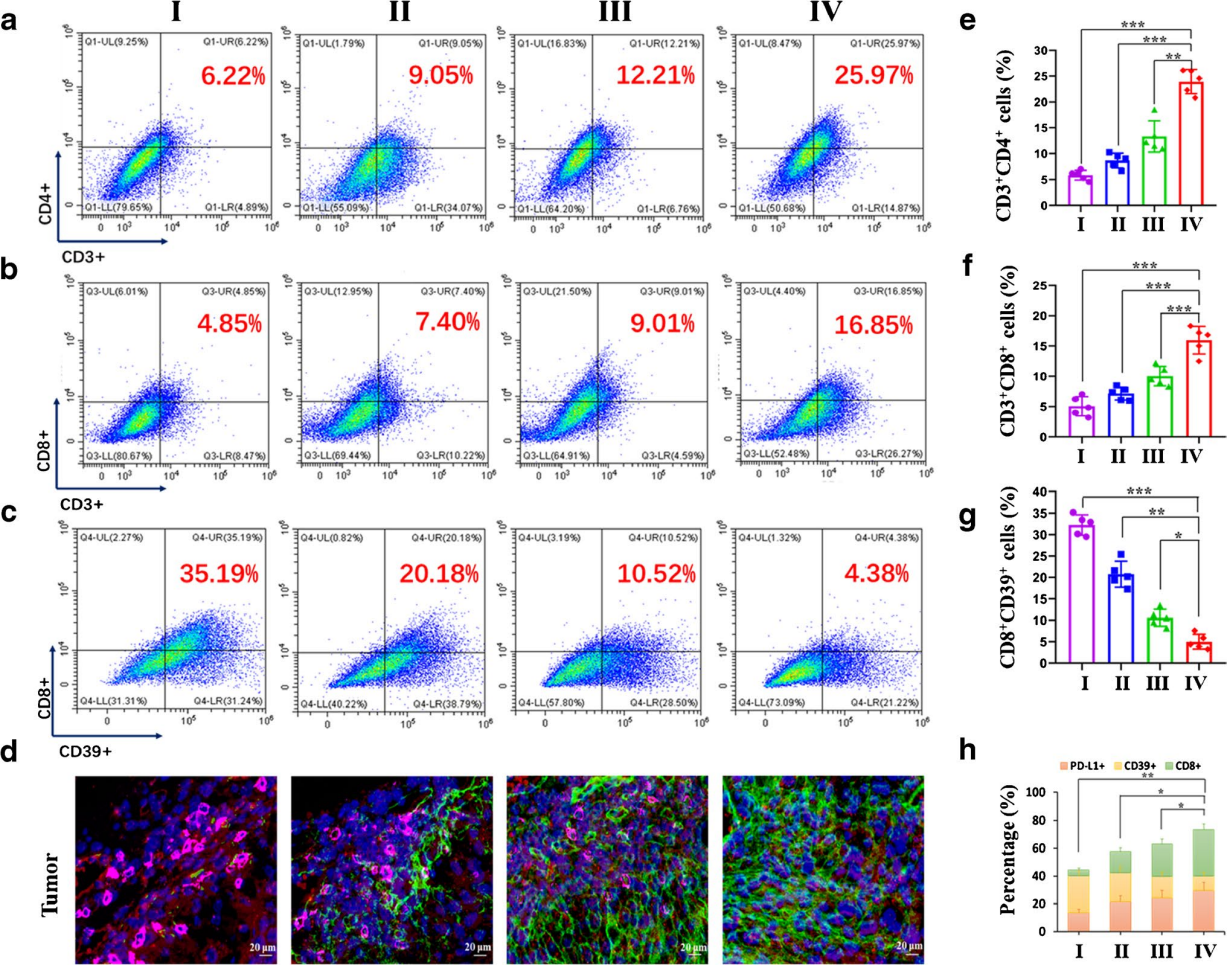
Percentage of tumor-infiltrating T cells (CD3^+^/CD4^+^, CD3^+^/CD8^+^) (**a**, **b**) and regulatory T cells (CD8^+^/CD39^+^) (**c**) in B16F10 tumors collected at the end of different treatments (group I, saline; group II, one dose of KN046 (1 mg); group III, one fraction of [^131^I]-KN046 ([^131^I]: 1 × 3.7 MBq, KN046: 1 mg); group IV, three fractions of [^131^I]-KN046 ([^131^I]: 1 × 3.7 MBq, KN046: 1 mg) at days 1, 4, and 7). **d** Representative multicolor immunofluorescence micrographs for PD-L1 (red), CD8^+^ (green), and CD39^+^ (pink) in B16F10 tumors collected at the end of different treatments. Cell nuclei are stained with DAPI (blue). Quantitative analysis of CD3^+^/CD4^+^ (**e**), CD3^+^/CD8^+^ (**f**), and CD8^+^/CD39^+^ (**g**) T cells in B16F10 tumors 3 days after different treatments. **h** Quantified results for multicolor immunofluorescence staining presented in Fig. 6d. Data are the mean ± SD (*n* = 5). ^*^p < 0.05, ^**^p < 0.01, ^***^p < 0.001

To study the change in the TME following [^131^I]-KN046 treatment, flow cytometry and multiplex immunofluorescence staining were used to assess changes in immunocytes. Flow cytometry (Fig. 6a, b, e, and f) showed a considerable antitumor response after KN046 therapy, with the percentages of CD4^+^ T cells (12.21% vs. 9.05%) and CD8^+^ T cells (9.01% vs. 7.40%) in the tumor increasing after treatment with [^131^I]-KN046 versus KN046 alone. Meanwhile, compared with the one-fraction [^131^I]-KN046 treatment group, the three-fraction [^131^I]-KN046 treatment group showed more CD4^+^/CD8^+^ T cells (*p* < 0.05). In addition, the average number of CD39^+^/CD8^+^ [34] cells having an immunosuppressive effect was significantly lower than that in the control group, and the percentages of CD39^+^/CD8^+^ cells in the group of treatment with three consecutive fractions of [^131^I]-KN046 were significantly lower (*p* < 0.05) (Fig. 6c and g). Multiplex immunofluorescence assay of tumor sections showed similar results (Fig. 6d and h). Also, the percentages of CD4^+^ T cells and CD8^+^ T cells in the spleen had the same trend (Fig. S5 a–f).

**Fig. 7:**
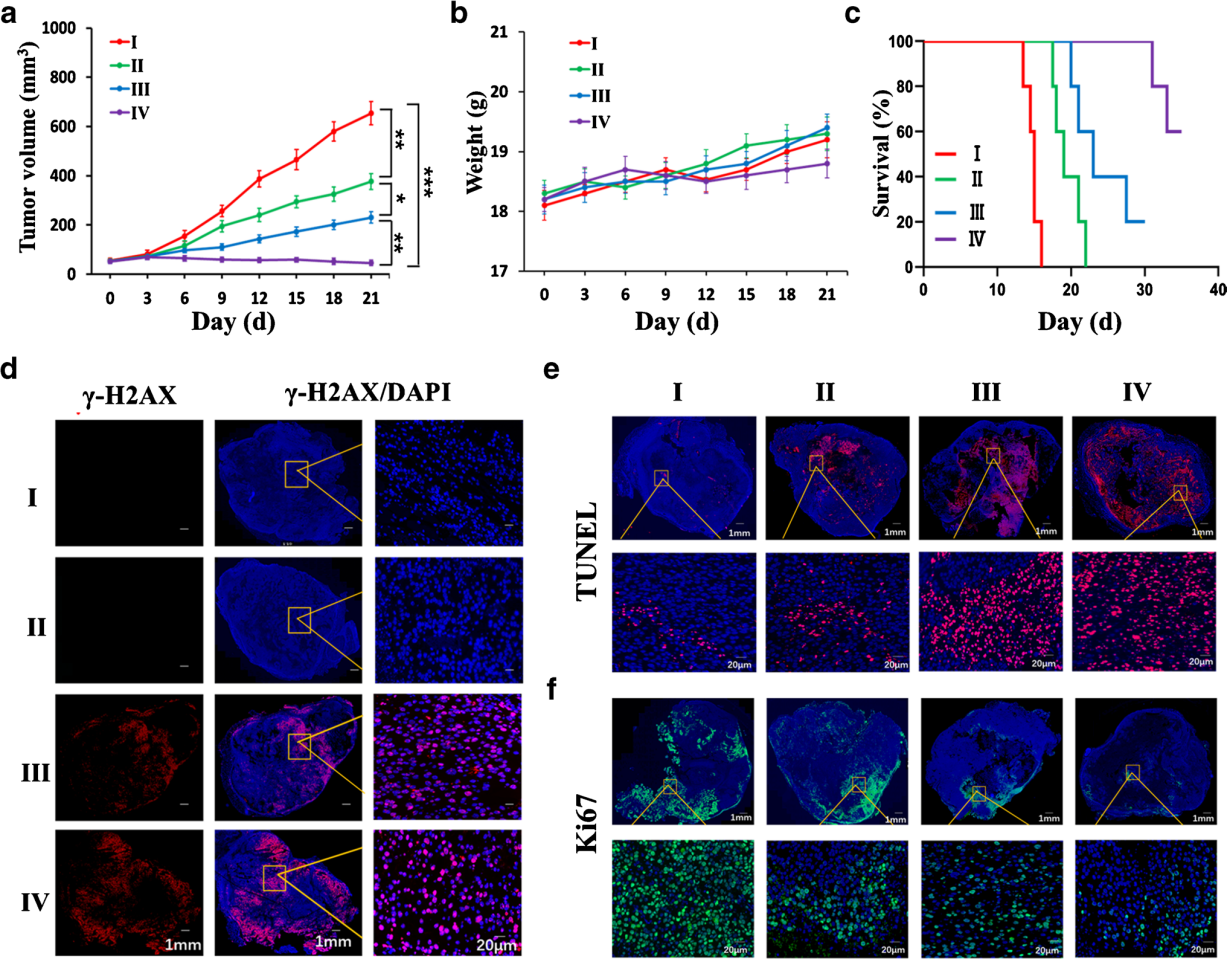
Tumor volume (**a**), bodyweight change (**b**), and survival (**c**) of mice bearing B16F10 tumors after different treatments (group I, saline; group II, one dose of KN046 (1 mg); group III, one fraction of [^131^I]-KN046 ([^131^I]: 1 × 3.7 MBq, KN046: 1 mg; group IV, three fractions of [^131^I]-KN046 ([^131^I]: 1 × 3.7 MBq, KN046: 1 mg) at days 1, 4, and 7). Representative multicolor immunofluorescence micrographs for γ-H2AX (**d**), TUNEL (**e**), and Ki67 (**f**) of B16F10 tumors from each group collected 3 days after different treatments. Cell nuclei are stained with DAPI (blue). Data are the mean ± SD (*n* = 5). ^*^p < 0.05, ^**^p < 0.01, ^***^p < 0.001

The residual tumor volume, viability, percent apoptosis of tumor tissue, and proliferation of Ki67 in tumors in mice were assessed to further validate the antitumor efficacy across different groups. In the KN046 treatment group and [^131^I]-KN046 treatment group, tumor growth was delayed to different extents. Over time, the primary tumor volume in the [^131^I]-KN046 group versus KN046 group shrank significantly (Fig. 7a). In B16F10 tumor-bearing mice receiving three-fraction [^131^I]-KN046 treatment, a significant difference in tumor volume was noted. [^131^I]-KN046 had good tolerability. The bodyweight of mice was relatively stable (Fig. 7b). Moreover, compared with KN046 therapy alone, treatment with [^131^I]-KN046 resulted in a pronounced increase in mouse survival (Fig. 7c). As shown by γ-H2AX and TUNEL staining (Fig. 7d and e), group IV exhibited the maximum area of DNA damage and the largest number of apoptotic cells compared with those in the three other groups, whereas the percentage of fluorescence-stained Ki67-positive cells was the smallest (Fig. 7f). Therefore, [^131^I]-KN046 achieved excellent antitumor efficacy, and maximum antitumor efficacy was observed in the three-fraction [^131^I]-KN046 treatment group (group IV).

### Safety of [.^131^I]-KN046

After [^131^I]-KN046 intervention at day 21, no abnormality was noted in mice with respect to hepatorenal functions and creatine kinase (Fig. S6a–c). Also, H&E sections of the liver, kidney, and heart indicated no established systemic biological toxicity upon [^131^I]-KN046 therapy (Fig. S6d–f).

## Discussion

In this study, we identified that systemic administration of [^131^I]-KN046 as a promising strategy noninvasively monitored PD-L1/CTLA-4 expression in vivo during an antitumor response with SPECT/CT, capable of increasing immunotherapy response rates and the survival rate of B16F10 tumor-bearing mice significantly.

Two major challenges in the immunotherapy for tumors come from the difficulties in identifying appropriate subset of patients respond [35] and reflecting the true status of the immune response [36, 37]. It is important to note that PD-L1 expression in the course of tumor progression/treatment is a process of dynamic change [38]. Hence, an IHC-based assay of PD-L1/CTLA-4 expression is invasive and perhaps not very accurate. It has been proven that molecular imaging can noninvasively identify the expression of immune checkpoint targets over time and monitor immunotherapeutic effects after the immunotherapy [39]. In this article, we demonstrated clearly that [^131^I]-KN046 had high affinity and specificity to two immune targets (PD-L1 and CTLA-4) and had excellent in vivo intratumoral retention so as to achieve good antitumor efficacy. Therefore, [^131^I]-KN046 can be used in SPECT/CT to reflect in vivo PD-L1/CTLA-4 expression in a real-time, quantitative, dynamic manner to determine the optimal “time window” of immunotherapy initiation. More importantly, granzyme B-targeted [^68^ Ga]-NOTA-GZP PET/CT enabled in vivo reflecting the efficacy of [^131^I]-KN046 therapy and the true status of the immune response. This phenomenon enabled noninvasive selection of the beneficiaries of PD-L1/CTLA-4 immunotherapy and identification of the non-responder or poor-responder patients to select “personalized therapy” as soon as possible.

A key to a synergistic antitumor strategy is to determine the best timing of combining radionuclide with immunotherapy to maximize efficacy [18, 40]. If immunosuppressant is carried out too late after radionuclide administration, it may not be benefit from the transient immune-activating effects of ionizing-radiation stimulus or miss the optimal time window for ICB immunotherapy. Chen et al. [18] reported that concurrent targeted radionuclide therapy (TRT) plus ICB therapy, rather than TRT followed by ICB therapy, was more effective for OS prolongation and long-term tumor control. In this regard, we also employed a concurrent treatment strategy in this study, and this scenario achieved the desired antitumor effect via a single administration of [^131^I]-KN046. Exposure of tumor cells to [^131^I]-KN046 led to upregulated expression of MHC-I and Fas surface molecules. Upregulation of expression of both proteins made tumor cells more susceptible to attack by the immune system, and after a modest dose of [^131^I] ionizing radiation, early DNA damage occurred in tumor cells. Therefore, the enhanced enrichment of low dose [^131^I] due to the targeting of KN046 disrupted the steady state of the TME. Meanwhile, the concurrent ICB antibody therapy will trigger and enhance the tumor-specific immune response.

Two other critical aspects need to be considered when developing new immunotherapy agents, including the increase of response rates [41] and the reduction of side effects. However, the objective response rate that responded to ICB immunotherapy is only 20 to 30% [41, 42]. As a recombinant PD-L1/CTLA-4 bispecific single-domain antibody Fc fusion protein, KN046 targets two immune checkpoints with a different mechanism, which could block the binding of PD-L1 to PD-1 and CTLA-4 to B7. Therefore, the uptake of [^131^I]-KN046 in B16F10 tumors was attributed to the high affinity and specificity to two immune targets (PD-L1 and CTLA-4) of KN046 rather than a specific enhanced permeability and retention effect (EPR) effects, which could improve antitumor efficacy and alleviate side effects significantly. In our animal model, antitumor efficacy after intravenous injection of [^131^I]-KN046 was obviously superior to that of KN046 monotherapy. Distinct antitumor superiority was proof that an ionizing-radiation stimulus mediated by an appropriate dose of [^131^I] was essential to immunopotentiation. This strategy enhanced the presentation of tumor antigens, boosted the sensitivity to the antitumor immune response, and reshaped the “hot” TME. Eventually, [^131^I]-KN046 enhanced tumor immunogenicity, resulting in significant increases in the degree of T-cell activation and counts of tumor-infiltrating immunocytes, and repeated therapy was more efficacious than a single intervention. Based on these advantages, a combination of low-dose [^131^I] and KN046 was synergistic with antitumor immunotherapy. This effect increased the number of tumor-infiltrating CTLs to yield strong and sustained antitumor efficacy, increase the immune response rate significantly, and extend OS.

To be honest, our study is limited to some extent, and whether [^131^I]-KN046 is capable of enhancing the sensitivity to immunotherapy in immunologically “cold” tumor model and other cancer models or not has to be further explored. And it is worth noting that concurrent injection is not always the best scenario with different drugs and cancer models. In the subsequent experiments, we will explore the dynamic time course of these observations across tumor cell types to determine the optimal timing and sequencing for immunotherapy intervention so as to choose the appropriate therapeutic protocols.

## Conclusion

We showed that systemic administration of [^131^I]-KN046 versus KN046 increased the immunotherapy response rates of B16F10 tumor-bearing mice. Mice could mediate elimination of tumor cells by the immune system more effectively, and the survival rate of mice increased significantly. A combination of low-dose [^131^I] and KN046 enhanced immunosensitivity significantly. Intravenous injection of [^131^I]-KN046 was synergistic with the antitumor immune response and exhibited strong antitumor efficacy while minimizing side effects and cost. We believe that this therapy has huge potential. Use of low-dose [^131^I] combined with a dual-target immunosuppressant was beneficial for increasing the immunotherapy response rate. This strategy will expand application of nuclear oncology in immunotherapies, provide a rationale for the clinical translation and efficacy assessment of [^131^I]-KN046, and create more opportunities for cancer treatment.

## Supplementary Information

Below is the link to the electronic supplementary material.

Supplementary file1 (DOCX 4221 KB)

## Data Availability

Original data are available on request.
