# Supplementary material for: Enhanced antitumor immune responses via a new agent [131I]-labeled dual-target immunosuppressant
Source: Eur J Nucl Med Mol Imaging. 2022 Oct 15;50(2):275–86. doi: 10.1007/s00259-022-05986-4 (PMC9816240; doi:10.1007/s00259-022-05986-4)
Supplement: Supplementary file 1 — Supplementary file1 (DOCX 4221 KB) [file 259_2022_5986_MOESM1_ESM.docx]

**Enhanced Antitumor Immune Responses via a New Agent [^131^I]-labeled** **Dual-target Immunosuppressant**

Chunjuan Jiang^1,2,3^, Qiwei Tian^4^, Xiaoping Xu^1,3^, Panli Li^1,3^, Simin He^1,3^, Jian Chen^5^, Bolin Yao^5^, Jianping Zhang^1,3^, Ziyi Yang^1,3^, Shaoli Song^1,3,^*

^1^ Department of Nuclear Medicine, Fudan University Shanghai Cancer Center, Shanghai, China

^2^ Department of Nuclear Medicine,The Second Xiangya Hospital,Central South University, Changsha, China.

^3^ Shanghai Engineering Research Center of Molecular Imaging Probes, Shanghai, China

^4^ Shanghai Key Laboratory of Molecular Imaging, Shanghai University of Medicine and Health Sciences, Shanghai, China

^5^ Key Laboratory of Smart Drug Delivery, Ministry of Education, School of Pharmacy, Fudan University, Shanghai, China

✉ Shaoli Song

shaoli-song@163.com

**MATERIALS AND METHODS**

**Reagents and instruments**

None of the chemicals we employed needed to be purified further. KN046 was purchased from Alphamab Oncology (Suzhou, China). [^131^I] ([NaI]) solution was supplied by Shanghai Xinke Pharmaceuticals (Shanghai, China). PBS, FBS, Iodogen, dimethyl sulfoxide (DMSO), sodium bicarbonate, sodium carbonate, 4% paraformaldehyde, 3% hydrogen peroxide, and 3% bovine serum albumin (BSA) were from MilliporeSigma (Burlington, MA, USA) or Shanghai Aladdin Biochemical Technology (Shanghai, China). The CCK-8 kit was purchased from Dojindo Chemical Technology (Kumamoto, Japan). Permeabilization solution and rapid blocking buffer were from Beyotime Institute of Biotechnology (Shanghai, China). Anti-γ-H2AX antibody and fluorescent secondary antibody were from Cell Signaling Technologies (Danvers, MA, USA). MHC-I-fluorescein isothiocyanate (FITC), Fas-FITC, CD3- Phycoerythrin (PE), CD4-FITC, CD8- peridinin chlorophyll protein (PerCP) and CD39- allophycocyanin (APC) were from BioLegend (San Diego, CA, USA). Anti-PD-L1, anti-caspase 3, TUNEL, and Ki67 were from Abcam (Cambridge, UK). 3,3'-diaminobenzidine (DAB) chromogen solution was from Dako (Copenhagen, Denmark).

The instruments we used were a Mini-Scan Radio-TLC Scanner (MiniGita Star; Raytest, Straubenhardt, Germany), microplate reader (Thermo Ascent; Thermo Scientific, Waltham, MA, USA), CytoFLEX flow cytometer (Beckman Coulter, Brea, CA, USA), confocal microscope (LSM 800; Zeiss, Wetzlar, Germany), autoradiography apparatus (Typhoon FLA 9500; GE Healthcare Life Sciences, Chicago, IL, USA), and gamma counter (SN-6110; Rihuan Optoelectronic Instruments; Shanghai Institute of Nuclear Research, Shanghai, China) for radiometry.

**Synthesis of [^131^I]-KN046 and assessment of its stability**

KN046 was labeled with [^131^I] using the catalyst Iodogen. [^131^I]-labeled KN046 was synthesized. Briefly, into an Eppendorf tube containing 20 μg of Iodogen was added an aqueous solution containing KN046 (250 μg to 1 mg). A solution of free [^131^I] was added rapidly, and the solution was allowed to stand at room temperature for 30 min for radiosynthesis. KN046 and [^131^I]-KN046 were analyzed by circular dichroism (CD) spectroscopy to determine the secondary structure of protein.

The *in vitro* stability of the labeling product, [^131^I]-KN046, was tested by co-culture with fetal bovine serum (FBS) or phosphate-buffered saline (PBS) (1:6, *v/v*) for 2 h，4 h，6 h，12 h，24 h，48 h and 72 h. The labeling yield and stability were determined by instant thin-layer chromatography.

**Culture of B16F10 and MCF-7 cells and creation of a melanoma model in mice**

A murine melanoma cell line (B16F10) and breast cancer cell line (MCF-7) were purchased from the Cell Resource Center within the Shanghai Institutes for Biological Sciences in the Chinese Academy of Sciences (Shanghai, China). Roswell Park Memorial Institute (RPMI) 1640 medium supplemented with 10% FBS, 100 U/mL penicillin, and 100 μg/mL streptomycin was used. Cells were cultured at 37°C in a cell incubator in an atmosphere of 5% CO_2_. PD-L1 expression in B16F10 cells and MCF-7 cells was determined by IHC staining.

Female BALB/c mice (specific pathogen-free; 18-20 g) were purchased from Shanghai Lingchang Biotech (Shanghai, China). All animal experiments were approved by the Animal Study Committee of Fudan University Shanghai Cancer Center (2019 Cancer Research JS-147) in Shanghai, China, and carried out in accordance with *Guidelines for the care and use of laboratory animals* (US National Institutes of Health, Bethesda, MD, USA).

**Cell Counting Kit (CCK)-8 assay**

The CCK-8 assay was used to assess the toxicity of [^131^I]-KN046 to B16F10 cells. First, B16F10 cells were plated onto a 96-well plate (five replicate wells, 1×10^4^ cells/well), allowed to grow adherently for 24 h, and then co-cultured with RPMI 1640 medium containing [^131^I]-KN046 (KN046: 20 μg，[^131^I]: 0, 0.185, 0.37, 0.74, 1.48, 2.22, 2.96, 3.7 MBq/mL) for 24 h. Next, CCK-8 reagent (10 μL) was added to the cell medium in each well and stained for 1–2 h. Finally, the microplate reader was used to determine the optical density (OD) at 450 nm. The percent OD for each group relative to that of the control group was calculated.

**Cellular uptake**

Onto a glass slide of a confocal dish, B16F10 and MCF-7 cells were pre-plated (~2×10^5^ cells/dish) and cultured for 24 h. Fluorescent FITC-labeled KN046 solution was added and co-culture allowed for 1 h and 4 h. After each co-culture run, the supernatant was removed, and the pellet was washed repeatedly with PBS thrice, followed by staining of cell nuclei with DAPI. Confocal laser scanning microscopy (CLSM) photography was undertaken to assess the distribution of KN046 on B16F10 and MCF-7 cells. Fluorescent FITC labeling of KN046 was conducted following a protocol. Briefly, a desired amount of KN046 solution was transferred to a 10 K ultrafiltration tube, centrifuged at 7500 × *g* for 10 min at room temperature, and then made up to 500 μL with sodium bicarbonate–sodium carbonate buffer (pH = 9). This action was followed by five cycles of centrifugation to result in KN046-buffer. Into 25 μL of FITC-DMSO solution (1 mg/mL) was added 500 μL of KN046-buffer, followed by thorough mixing, protection from light, and the reaction allowed to proceed overnight at 4°C. Finally, KN046-FITC solution in the 10 K ultrafiltration tube was centrifuged at 7500 × *g* for 10 min at room temperature five times, made up to 500 μL with ultrapure water, and protected from light at 4°C.

**Cellular gamma histone 2AX (γ-H2AX) assay**

First, onto a glass slide of a confocal dish, B16F10 cells were pre-plated (~2×10^5^ cells/dish), allowed to grow adherently for 24 h, and then co-cultured with 1 mL of RPMI 1640 medium spiked with 0.9% saline, KN046 (100 μg), [^131^I]-KN046 ([^131^I]: 1×1.85 MBq; KN046: 100 μg) or [^131^I]-KN046 ([^131^I]: 3×1.85 MBq; KN046: 3×100 μg) for 24 h. Second, B16F10 cells were fixed with 1 mL of PBS containing 4% paraformaldehyde for 10 min, and blocked with the permeabilization solution and rapid blocking buffer for 10 min at room temperature. Third, B16F10 cells were stained with anti-γ-H2AX antibody (1:400 dilution) and stored overnight at 4°C. Fourth, fluorescent secondary antibody (Alexa Fluor^®^ 555)-labeled goat anti-rabbit IgG (1:400 dilution) was added, and incubation away from light for 1 h at room temperature permitted. Thereafter, the nuclei of B16F10 cells were stained with DAPI. Finally, B16F10 cells were photographed using the fluorescence confocal microscope at an excitation wavelength of 565 nm.

**Flow cytometry of MHC-I and Fas in cells**

Studies have shown that radiation at a sublethal dose leads to upregulation of expression of several types of molecules on tumor-cell surfaces, including Fas and MHC-I. To ascertain if exposure to [^131^I]-KN046 had the same effect, B16F10 cells were pre-plated onto a six-well plate (~1×10^5^ cells/well), allowed to grow adherently for 24 h, and then co-cultured with 2 mL of RPMI 1640 medium spiked with 0.9% saline, KN046 (100 μg), [^131^I] (1.85 MBq), [^131^I]-KN046 ([^131^I]: 1×1.85 MBq; KN046: 100 μg), or [^131^I]-KN046 ([^131^I]: 3×1.85 MBq; KN046: 3×100 μg) for 24 h. Thereafter, cells were washed and plated onto a new six-well plate for incubation for 24 h. Then, the resulting cells were harvested, spiked with a fluorescent FITC-labeled antibody against cell-surface molecules (MHC-I and Fas), and incubated away from light for 40 min at 4°C. Finally, cell samples were loaded for flow cytometry to analyze surface molecules.

**SPECT/CT**

SPECT/CT was carried out using a small animal-specific SPECT/CT scanner (nanoScan@SC SPECT/CT 4 Detector; Mediso Medical Imaging Systems, Budapest, Hungary).

Mice were given potassium iodide (KI) solution (0.5~0.8 g/mL) 7 days before SPECT/CT imaging in order to reduce the uptake of free iodine by the thyroid tissue.After [^131^I]-KN046 ([^131^I]: 7.4 MBq, KN046: 100 μg) had been injected *via* the caudal vein in B16F10 tumor-bearing mice, systemic SPECT/CT images were captured at 6 h, 24 h, 48 h, 72 h, 5 d, 7 d, and 10 d, respectively(n = 5 per group). Each CT scan (50 kV, 980 μA) of 6-min duration was followed by one SPECT/CT acquisition of 20–40 min (matrix: 128 × 128, frame time: 30–60 s). After completion of acquisition, SPECT/CT data were reconstructed using the TeraTomo 3D (TT3D) dynamic range. SPECT/CT images were analyzed using Nucline 3.00 (Mediso Medical Imaging Systems). Regions of interest were plotted and contralateral front-limb muscles were chosen as the background of SPECT/CT images. Results were expressed as the target-to-normal tissue ratios (TNR).

For SPECT/CT for the blockade group, an overdose of KN046 (5 mg) was injected in B16F10 tumor-bearing mice *via* the caudal vein. Then, 30-min later, [^131^I]-KN046 ([^131^I]: 7.4 MBq; KN046: 100 μg) was injected. Finally, systemic SPECT/CT images were captured at 24, 48, and 72 h, respectively.

For SPECT/CT for the control group, after [^131^I]-KN046 ([^131^I]: 7.4 MBq, KN046: 100 μg) had been injected in MCF-7 tumor-bearing mice *via* the caudal vein, systemic SPECT/CT images were captured at 24, 48, and 72 h, respectively.

***In vitro* autoradiography**

To validate the accuracy of SPECT/CT data in the assay of tumor uptake of [^131^I]-KN046, tumor tissues were excised for autoradiography. At each corresponding time point of SPECT/CT, the mice were killed(n = 5 per group). The tumor tissues were made into frozen sections and tabletted on the phosphor screen for 24 h. By scanning the phosphor screen using Typhoon 2.0 (GE Healthcare), images were captured, and the quantified data were analyzed using ImageQuant TL 8.1 (GE Healthcare). Meanwhile, H&E was used to stain tumor tissues, and the images acquired were compared with those captured by autoradiography.

**Pharmacokinetics and biodistribution of [^131^I]-KN046**

An appropriate dose of [^131^I]-KN046 ([^131^I]: 0.74 MBq; KN046: 100 μg) was injected in normal BALB/c mice *via* the caudal vein (n = 5 per group). At prescribed time points (15 min, 30 min, 1 h, 2 h, 4 h, 6 h, 1 d, 2 d, 3 d, 4 d, 5 d, 6 d, and 7 d), 10 μL of caudal-vein blood was collected with a capillary each time, weighed, and measured with a gamma counter for the radiometric count rate. The measured activity was expressed in radioactivity per liter of blood (MBq/L). Using Prism 9.2 (GraphPad, San Diego, CA, USA), a nonlinear regression curve was fitted to calculate pharmacokinetic parameters.

Seven days after inoculation with B16F10 cells, [^131^I]-KN046 ([^131^I]: 0.74 MBq; KN046: 100 μg) was injected *via* the caudal vein for biodistribution studies(n = 5 per group). At prescribed time points (6, 24, 48, and 72 h), tumors as well as selected tissues and organs were harvested, weighed, and counted using the gamma counter. The results were listed as percent injected dose per gram of tissue (%ID/g).

***In vivo* treatment**

In the mouse model of melanoma, 3×10^5^ B16F10 cells were engrafted by subcutaneous injection at the right armpit. Tumor size was determined using a vernier caliper. Tumor volume was calculated using the formula volume = (length × width^2^)/2. When tumor volume reached ~60 mm^3^, mice were randomized into groups Ⅰ, Ⅱ, Ⅲ, and Ⅳ, respectively (day-1 of treatment). Then, an effective therapeutic dose of [^131^I]-KN046 was injected *via* the caudal vein (n = 5 per group).They received 0.9% saline, one dose of KN046 (1 mg), one fraction of [^131^I]-KN046 ([^131^I]: 1×3.7 MBq; KN046: 1 mg), and three fractions of [^131^I]-KN046 ([^131^I]: 1×3.7 MBq; KN046: 1 mg), respectively, at days 1, 4, and 7. All doses were given in 100 µL (total volume) of 0.9% saline diluent by injection *via* the caudal vein. Bodyweight and tumor size were measured once every 3 days.

**[^18^F]-fluorodeoxyglucose micro-positron emission tomography/computed tomography ([^18^F]-FDG micro-PET/CT) and [^68^Ga]-NOTA-GZP (granzyme B-specific PET imaging agent) micro-PET/CT imaging**

Micro-PET/CT was done using an Inveon Animal-PET/CT Scanner (Siemens Preclinical Solutions, Knoxville, TN, USA). For the tumor-bearing mice in group Ⅳ, [^18^F]-FDG micro-PET/CT and [^68^Ga]-NOTA-GZP micro-PET/CT were conducted at 0, 3, 6, 9, and 12 d(n = 5 per group). Before [^18^F]-FDG micro-PET/CT, mice were fasted and deprived of water for 6 h, administered [^18^F]-FDG (7.4 MBq, 100 μL) by injection *via* the caudal vein, kept warm, and anesthetized with low-concentration isoflurane gas for 1 h before imaging. [^68^Ga]-NOTA-GZP (7.4 MBq, 150 μL) was injected in each mouse *via* the caudal vein 1 h before [^68^Ga]-NOTA-GZP micro-PET/CT. During imaging, each mouse was anesthetized with a mixture of isoflurane and oxygen gas lying prone on the scanning bed. Continuous acquisition of CT images for 5 min was conducted using Inveon Acquisition 2.0.0.10, followed by a 10-min static PET scan. Acquired images were reconstructed automatically *via* three-dimensional ordered-subset expectation maximization (OSEM3D) and processed and analyzed on Inveon Research Workplace. Tumors were delineated and tumor SUV_max_ was measured, with normal liver as the background of [^18^F]-FDG micro-PET/CT images and contralateral front-limb muscle as the background of [^68^Ga]-NOTA-GZP micro-PET/CT images, and the TNR was calculated.

**Flow cytometry of immunocytes**

Three days after treatment completion in each group, tumors and spleen tissues in all mice were harvested, and cut into chips (about 1–2 mm in size, n = 5 per group). A round syringe handle was held to squeeze the chips gently onto a 200-mesh cell filter. We filtered the cell suspension, and the filtrate was centrifuged at 350 × *g* for 5 min at room temperature. Then, after anti-cell-surface-molecule antibodies (CD3-PE, CD4-FITC, CD8-cy5.5, and CD39-APC) had been added, respectively, the filtrate was incubated away from light for 40 min at 4°C. The CytoFLEX flow cytometer was used to characterize cells and search for tumor-infiltrating T cells in tumor tissues. The results of flow cytometry were analyzed using FlowJo (Becton Dickinson, Ashland, OR, USA) and CD3^+^ cells were gated to identify immunocytes. CD8^+^ T cells were identified as CD3^+^/CD8^+^. CD4^+^ T cells were identified as CD3^+^/CD4^+^. Regulatory T cells were identified as CD3^+^/CD8^+^/CD39^+^.

**Immunohistochemical (IHC) staining and immunofluorescence staining**

Each tumor tissue was fixed with 4% paraformaldehyde and embedded in a paraffin block, and then made into a paraffin sections (thickness = 5 μm) for IHC staining(n = 5 per group). For the latter, the paraffin section was deparaffinized and rehydrated, incubated away from light in 3% hydrogen peroxide solution at room temperature for 25 min, and blocked with 3% BSA for 30 min at room temperature. Then, anti-PD-L1 (1:200 dilution; Abcam) was added dropwise into the wet box, followed by incubation overnight at 4°C. Next, a desired volume of horseradish peroxidase-labeled secondary antibody working solution was added dropwise, followed by incubation for 50 min at 37°C. Subsequently, DAB chromogen solution was added dropwise. Images were captured by a microscope.

For immunofluorescence staining, the paraffin section was first deparaffinized and rehydrated. Then, the resulting tissue section was placed in a retrieval box fully loaded with EDTA antigen retrieval buffer (pH 8.0) and put into a microwave oven for antigen retrieval. After incubation of the section in BSA for 30 min, antibodies against caspase-3, anti-γ-H2AX, terminal deoxynucleotidyl transferase dUTP nick end labeling (TUNEL), and Ki67 were added dropwise into a wet box, followed by incubation overnight at 4°C. Next, the corresponding horseradish peroxidase-labeled secondary antibodies were added dropwise, followed by incubation for 50 min at 37°C. For syngeneic dual-labeled or triple-labeled immunofluorescence staining, the second (third) primary antibody and secondary antibody working solution were further added following antigen retrieval. Finally, cell nuclei were counterstained with DAPI. The section was mounted with anti-quenching mounting medium and then photographed under a fluorescence microscope.

***In vivo* safety**

[^131^I]-KN046 ([^131^I]: 1×3.7 MBq; KN046: 1 mg) was injected in normal mice *via* the caudal vein (n = 5 per group). Vital signs were observed for 21 consecutive days, then the mice were killed for assay of hepatorenal functions as well as H&E staining of the heart, liver, spleen, lungs, and kidneys.

**Statistical analyses**

Statistical analyses were carried out using SPSS 24.0 (IBM, Armonk, NY, USA). Data are the mean ± standard deviation. The Student’s *t*-test was used to compare data from two samples. One-way ANOVA was employed to assess multiple between-group variations. Kaplan–Meier curves and log-rank (Mantel–Cox) tests were used to analyze mouse viability. All graphs were considered statistically significant when ^*^p < 0.05, ^**^p < 0.01, ^***^p < 0.001.

**Results**


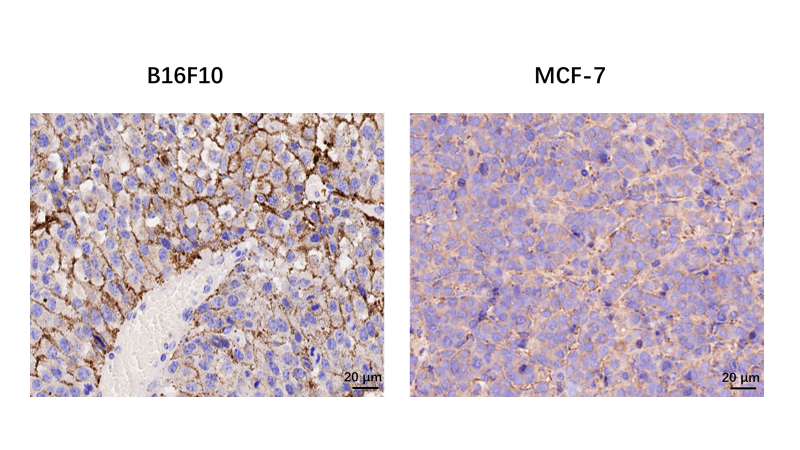


**Fig. S1**  Immunohistochemical (IHC) staining for PD-L1. Brown staining on the cell membrane indicates PD-L1^+^ cells. Expression of PD-L1 protein was strongly positive in B16F10 cells (left) and negative in MCF-7 cells (right).


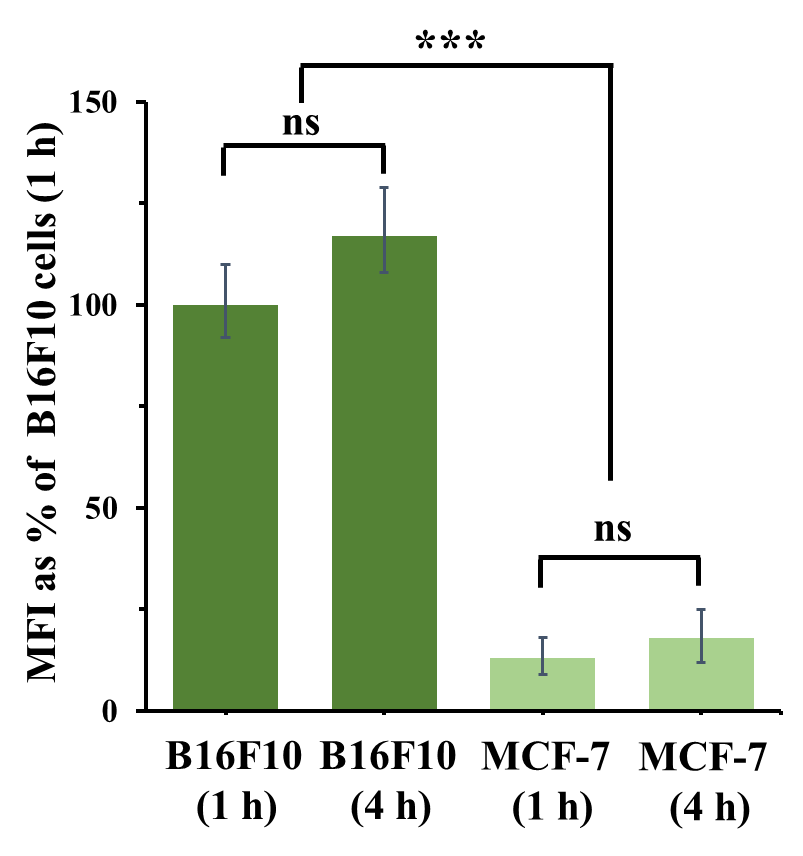


**Fig. S2** Quantitative analysis of KN046 mean fluorescence intensity (MFI) on B16F10 and MCF-7 cells in fluorescent microscopy images at 1 h and 4 h. Data are the mean ± SD (n = 5). ^***^p < 0.001, ns=no significance.


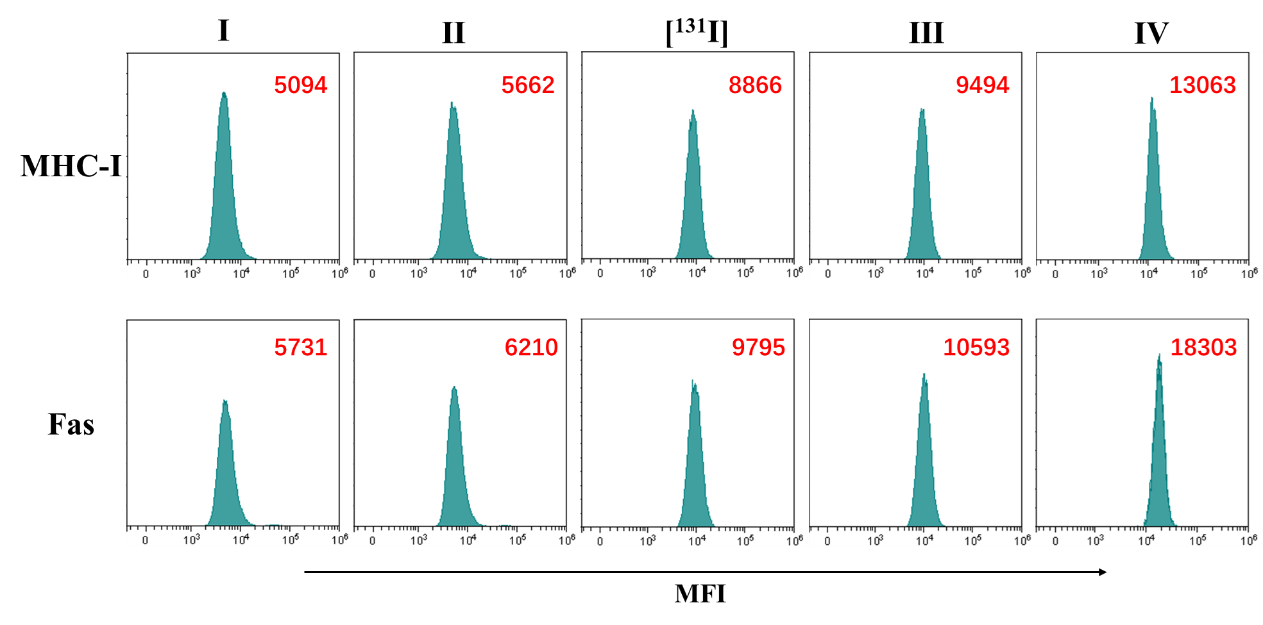


**Fig. S3**  Expression of MHC-I (top row) and Fas (bottom row) on the B16F10 cell surface after different treatments, respectively[Group Ⅰ: saline, Group Ⅱ: one dose of KN046 (100 μg), [^131^I]: (1.85 MBq), Group Ⅲ: one fraction of [^131^I]-KN046 ([^131^I]: 1×1.85 MBq; KN046: 100 μg), Group Ⅳ: three fractions of [^131^I]-KN046 ([^131^I]: 3×1.85 MBq; KN046: 3×100 μg).]. MFI, mean fluorescence intensity.


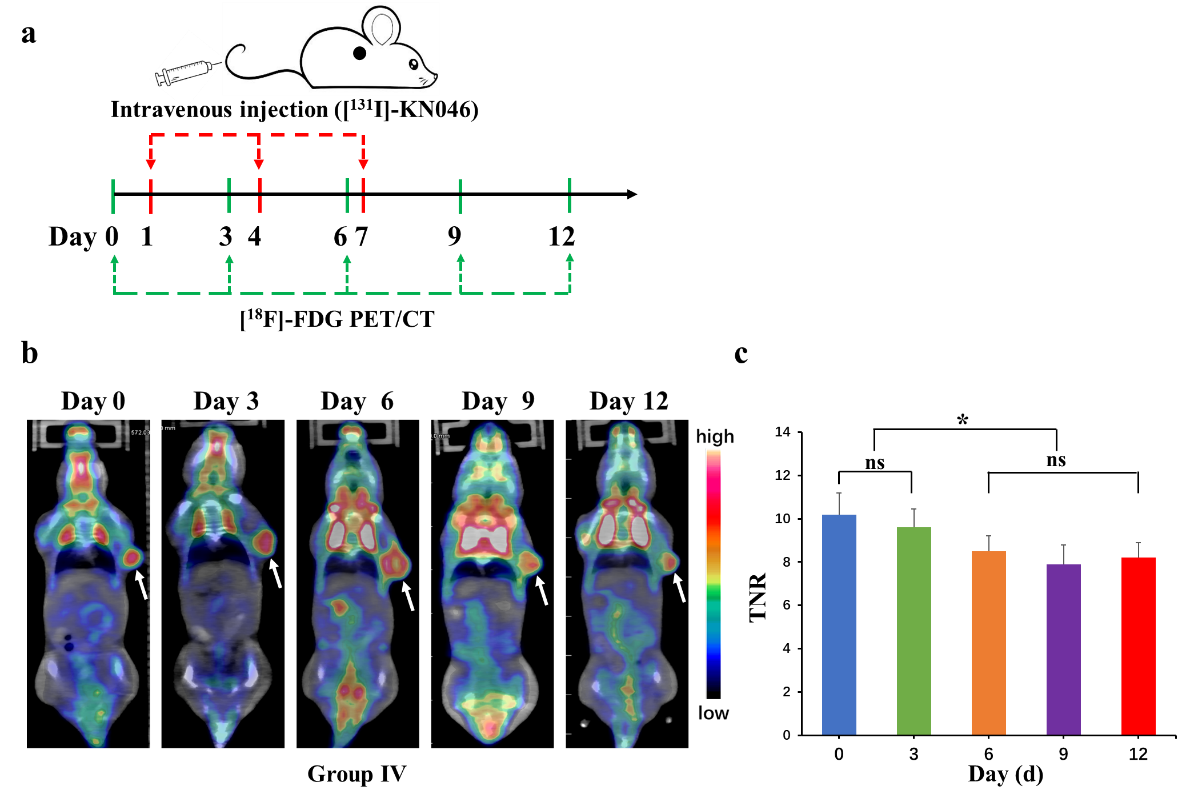


**Fig. S4**  (a) Therapy protocol and [^18^F]-FDG micro-PET/CT in mice bearing B16F10 tumors (schematic) [Group Ⅳ: three fractions of [^131^I]-KN046 ([^131^I]: 1×3.7 MBq, KN046: 1 mg) at days 1, 4, and 7. [^18^F]-FDG micro PET/CT were performed at days 0, 3, 6, 9, and 12.].(b) Representative [^18^F]-FDG micro-PET/CT images in mice bearing B16F10 tumors at different times in group Ⅳ. White arrows denote tumors.(c) TNR quantitative analysis of the images in B16F10 tumors collected 3 days after treatment of group Ⅳ. Data are the mean ± SD (n = 5). *p < 0.05, ns:no significance.


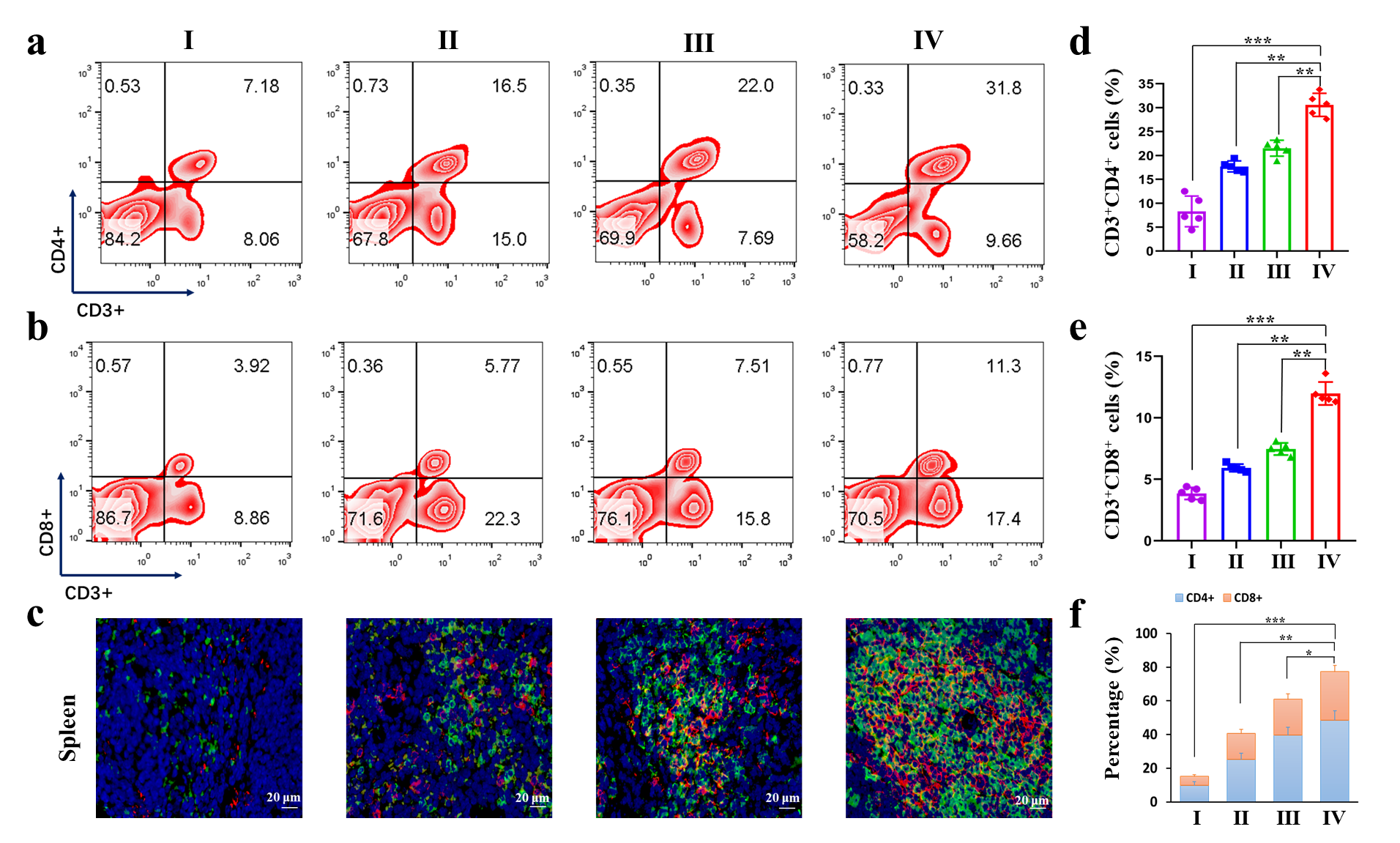


**Fig. S5** (a) Percentage of CD3^+^/CD4^+^ (a) and CD3^+^/CD8^+^ T cells (b) in spleens from mice bearing B16F10 tumors at the end of different treatments[Group Ⅰ: saline, Group Ⅱ: one dose of KN046 (1 mg), Group Ⅲ: one fraction of [^131^I]-KN046 ([^131^I]: 1×3.7 MBq, KN046: 1 mg), Group Ⅳ: three fractions of [^131^I]-KN046 ([^131^I]: 1×3.7 MBq, KN046: 1 mg) at days 1, 4, and 7.].(c) Representative multicolor immunofluorescence micrographs for CD4^+^ (green) and CD8^+^ (red) of spleens from mice bearing B16F10 tumors collected at the end of different treatments. Cell nuclei are stained with DAPI (blue).Quantitative analysis of CD3^+^/CD4^+^ (d) and CD3^+^/CD8^+^ (e) in spleens from mice bearing B16F10 tumors 3 days after different treatments.(f) Quantified results for multicolor immunofluorescence staining presented in Fig. S6c.Data are the mean ± SD (n = 5). *p < 0.05, **p < 0.01, ***p < 0.001.


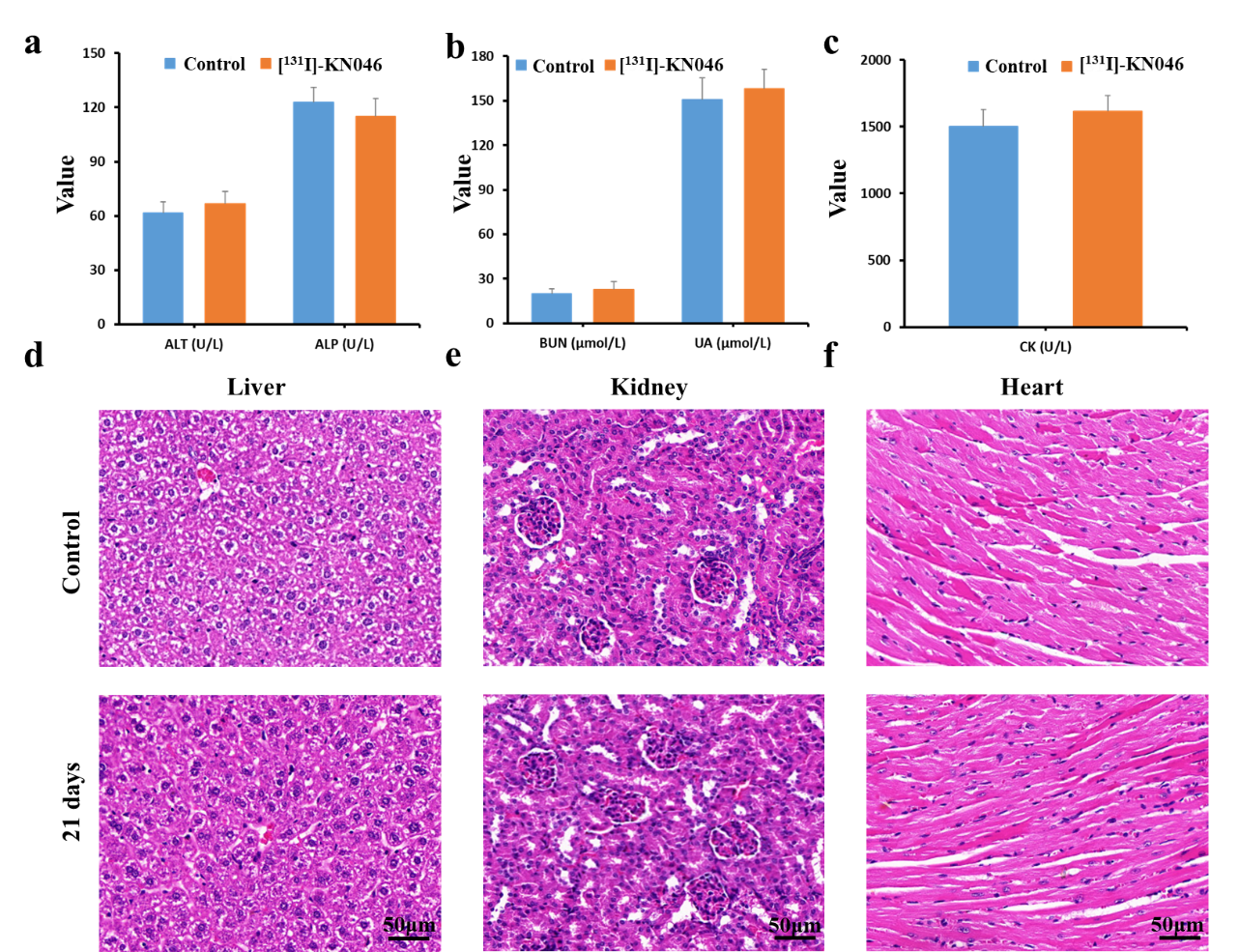


**Fig. S6** Analyses of function of the liver (a), kidney (b) and heart (c) of control and experiment groups for evaluation of [^131^I]-KN046 toxicity. (d) H&E staining of the major organs from normal mice in control and experimental groups at 21 d post-injection. Data are the mean ± SD (n = 5).
